# Supplementary material for: Spontaneously formed phonon frequency combs in van der Waals solid CrGeTe3 and CrSiTe3
Source: Nat Commun. 2025 Jul 1;16:5795. doi: 10.1038/s41467-025-61173-7 (PMC12216165; doi:10.1038/s41467-025-61173-7)
Supplement: Supplementary file 1 — Supplementary Information [file 41467_2025_61173_MOESM1_ESM.pdf]

Supplementary Information for Spontaneously formed phonon frequency combs in van der Waals solid $CrGeTe_3$ and $CrSiTe_3$

I. Calculation of anharmonic oscillators

The Hamiltonian for anharmonic oscillators is

$$H = \frac{p^2}{2\mu} + \frac{1}{2}\mu\omega^2 x^2 + \lambda x^3, \tag{1}$$

and we denote the harmonic eigenstates as $|n\rangle$. It is easy to see that the first-order perturbation energy for every eigenstate $\langle n|\lambda x^3|n\rangle$ is zero since the cubic term contains an odd number of operators. Using second-order perturbation theory, we can get the modified energy levels

$$E_n = \hbar\omega(n + \frac{1}{2}) - \frac{1}{8}\frac{\lambda^2\hbar^2}{\mu^3\omega^4}[30(n + \frac{1}{2})^2 + \frac{7}{2}] \tag{2}$$

with

$$E_{n+1} - E_n = \hbar\omega - \frac{15}{2}\frac{\lambda^2\hbar^2}{\mu^3\omega^4}(n+1) \tag{3}$$

Denoting $A = \frac{15}{2}\frac{\lambda^2\hbar}{\mu^3\omega^4}$, we get

$$E_{n+1} - E_n = \hbar\omega - \hbar A(n+1), \tag{4}$$

and $A \geq 0$.

We then calculate the effect of this energy modulation on the time evolution of x in the coherent state. The coherent state is written as

$$|\alpha_0\rangle = e^{-\frac{|\alpha_0|^2}{2}} \sum_{n=0}^{\infty} \frac{\alpha_0^n}{\sqrt{n!}}|n\rangle \tag{5}$$

and its time evolution is

$$|\alpha_0\rangle_t = e^{-\frac{|\alpha_0|^2}{2}} \sum_{n=0}^{\infty} \frac{\alpha_0^n}{\sqrt{n!}} e^{-iE_n t/\hbar}|n\rangle. \tag{6}$$

Using this, we can calculate the time evolution of the annihilation operator $\langle a \rangle$:

$$\begin{aligned}\langle a\rangle_t =&\langle \alpha_0^* | a | \alpha_0 \rangle_t \\ =&e^{-|\alpha_0|^2} \sum_{k=0}^{\infty} \sum_{n=1}^{\infty} \frac{\alpha_0^{*k}}{\sqrt{k!}} \frac{\alpha_0^n}{\sqrt{(n-1)!}} e^{-i(E_k - E_n)t/\hbar} \langle k | n-1 \rangle \\ =&e^{-|\alpha_0|^2} \sum_{n=0}^{\infty} \frac{|\alpha_0|^{2n} \alpha_0^*}{n!} e^{i(E_{n+1}-E_n)t/\hbar} \\ =&e^{-|\alpha_0|^2} \sum_{n=0}^{\infty} \frac{|\alpha_0|^{2n} \alpha_0}{n!} e^{-i(\omega - A(n+1))t} \\ =&\alpha_0 e^{-|\alpha_0|^2 - i(\omega - A)t} \sum_{n=0}^{\infty} \frac{|\alpha_0|^{2n}}{n!} e^{inAt},\end{aligned} \tag{7}$$

where we used $\langle k|n-1\rangle = \delta(k, n-1)$ and $E_{n+1} - E_n = \hbar\omega - \hbar A(n+1)$. Notice that $\sum_{n=0}^{\infty} \frac{|\alpha_0|^{2n}}{n!} e^{inAt}$ is a Taylor series expansion of $\exp(|\alpha_0|^2 e^{iAt})$, therefore we get

$$\begin{aligned}\langle a\rangle_t =&\alpha_0 e^{-|\alpha_0|^2 - i(\omega - A)t} \exp(|\alpha_0|^2 e^{iAt}) \\ =&\alpha_0 \exp[-i(\omega - A)t + |\alpha_0|^2 (e^{iAt} - 1)],\end{aligned} \tag{8}$$

which leads to the equation (6) in the main text. A few sanity checks can be made here, for example, for $A = 0$, the equation changes to the ordinary $e^{-i\omega t}$ oscillation, and the change of α_0 will not make a difference in the oscillation frequency. For nonzero A, at small α_0 the $|\alpha_0|^2(e^{iAt} - 1)$ term is less pronounced, indicating that the nonlinear effect is only visible at larger oscillation amplitude, which is also consistent with one's impression on nonlinear optics.

The Raman process is based on the polarizability change of the system as a function of the atomic displacement, which to the first order

$$\beta = \beta_0 + \left(\frac{\partial \beta}{\partial x} \right)_0 x \tag{9}$$

here β is the polarizability, with β_0 being the polarizability at the equilibrium position, and we use x instead of $\langle x \rangle$ for simplicity. If we assume an incident light wave with electric field $E = E_0 \cos(\omega_\nu t)$ where E_0 is the amplitude and ω_ν is the photon frequency, from the expression of induced dipole moment $\mu = \beta E$, one can get

$$\mu = \beta_0 E_0 \cos(\omega_\nu t) + \left(\frac{\partial \beta}{\partial x} \right)_0 x(t) E_0 \cos(\omega_\nu t) \tag{10}$$

where the first term correspond to the Rayleigh scattering, and the second term is Raman scattering, denoted as μ_{R}. A Fourier transform of μ_{R} into the frequency space

gives

$$\begin{aligned}\mu_{\mathrm{R}}(\omega) &= \left(\frac{\partial \beta}{\partial x}\right)_0 E_0 \int_{-\infty}^{\infty} x(t)\cos(\omega_\nu t) e^{-i\omega t} dt \\ &= \left(\frac{\partial \beta}{\partial x}\right)_0 E_0 \left(\int_{-\infty}^{\infty} x(t) e^{-i\omega t} dt\right) * \left(\int_{-\infty}^{\infty} \cos(\omega_\nu t) e^{-i\omega t} dt\right) \\ &= \left(\frac{\partial \beta}{\partial x}\right)_0 E_0 \pi \left(x(\omega) * [\delta(\omega - \omega_\nu) + \delta(\omega + \omega_\nu)]\right)\end{aligned} \tag{11}$$

Where the convolution theorem is used and $*$ denotes convolution. This equation indicates that the induced diple moment has the shape of $x(\omega)$ convoluted with a delta function centered at the incident photon frequency ω_ν. In the Raman spectrum shown in this work, the delta function convolution is considered and the the energy is set to zero at ω_ν, therefore since the Raman intensity is directly proportional to the square of the induced moment $\mu_{\mathrm{R}}(\omega)$, we can get the conclusion in the main text that the Raman intensity is proportional to $\langle x\rangle_\omega^2$.

II. Raman tensor and selection rules of $CrGeTe_3$

Without magnetism, the space group of $CrGeTe_3$ gives 10 Raman active modes, including five E_{g} modes and five A_{g} modes. The E_{g} modes are doubly degenerate and can be further split into $E_{1\mathrm{g}}$ and $E_{2\mathrm{g}}$ modes. The Raman tensor of these modes can be written as[1]:

$$A_{\mathrm{g}} = \begin{bmatrix} a & 0 & 0 \\ 0 & a & 0 \\ 0 & 0 & b \end{bmatrix}, E_{1\mathrm{g}} = \begin{bmatrix} c & d & e \\ d & -c & f \\ e & f & 0 \end{bmatrix}, E_{2\mathrm{g}} = \begin{bmatrix} d & -c & -f \\ -c & -d & e \\ -f & e & 0 \end{bmatrix} \tag{12}$$

The Raman intensity can then be calculated using the equation

$$I = |\phi^\dagger R \phi|^2 \tag{13}$$

with $\phi = \frac{1}{\sqrt{2}}\begin{bmatrix} 1 \\ i \\ 0 \end{bmatrix}$ and $\frac{1}{\sqrt{2}}\begin{bmatrix} 1 \\ -i \\ 0 \end{bmatrix}$ for left-hand and right-hand polarizations, respectively. Replacing the Raman tensor R with the specific Raman tensor shown in eqn. (12), one can get the Raman intensity for each polarization as shown in TABLE. I, which indicates that the E_{g} and A_{g} modes are only respectively active in cross-polarization and parallel-polarization channels for circularly polarized lights.

III. Temperature dependence of phonon energy

To the lowest order the phonon energy change can be attributed to two sources:

$$\Delta E = \Delta E_{\mathrm{s}} + \Delta E_{\mathrm{t}} \tag{14}$$

Polarization	A_{g}	$E_{1\mathrm{g}}$	$E_{2\mathrm{g}}$
LR	0	c^2+d^2	c^2+d^2
LL	a^2	0	0
RL	0	c^2+d^2	c^2+d^2
RR	a^2	0	0

Supplementary Table 1: Raman intensity calculated from the Raman tensor of $CrGeTe_3$.

where ΔE_{s} comes from anharmonic decay calculated from a damped harmonic oscillator model

$$\Delta E_{\mathrm{s}} = \sqrt{E_0^2 - \Gamma^2} - E_0 \approx -\frac{\Gamma^2}{2E_0} \tag{15}$$

for small values of $\frac{\Gamma}{E_0}$. ΔE_{t} is from thermal expansion

$$\begin{aligned} \Delta E_{\mathrm{t}}(T) &= E_0 \left\{ \exp\left[-\gamma_i \int_0^T \beta(T')\, dT' \right] - 1 \right\} \\ &\approx E_0 \left\{ \exp\left[-\gamma_i \frac{V(T)-V(0)}{V(0)} \right] - 1 \right\} \\ &\approx -\gamma_i E_0 \frac{V(T)-V(0)}{V(0)} \end{aligned} \tag{16}$$

where $\beta(T) = \frac{1}{V}\frac{\partial V}{\partial T}$ is the volumetric thermal expansion coefficient and $\gamma_i = \frac{d\ln(E_i)}{d\ln(V)}$ is the Grüneisen parameter for the i-th phonon mode. This equation can be derived from integrating the definition equations of $\beta(T)$ and γ_i, and the last two lines hold only for small $\frac{V(T)-V(0)}{V(0)}$ values, which is less than 0.01 in our case.
For phonon softening from anharmonic decays, we can estimate from the phonon width that at 300K the upper limit of softening $\Delta E_{\mathrm{s}} = -\frac{\Gamma^2_{300\mathrm{K}}}{2E_0} \approx 0.03\ \mathrm{cm}^{-1}$, which is significantly lower than the observed $\Delta E = 2.7\mathrm{cm}^{-1}$, inferring that most phonon softening comes from the thermal expansion of the lattice. A complete temperature dependence of the cell volume of $CrGeTe_3$ has been reported[2], and if we assume a constant γ_i, from the fit shown in fig.3 we can get $\gamma_i = 1.65 \pm 0.08$ for the 532-nm data. A rough comparison can be made from the macroscopic Grüneisen parameter γ_{macro}:

$$\gamma_{\mathrm{macro(T)}} = \frac{\beta(T)K(T)V_{\mathrm{mol}}(T)}{C^p_{\mathrm{mol}}(T)} \tag{17}$$

where $K(T)$ is the bulk modulus, $V_{\mathrm{mol}}(T)$ is the molar volume, and $C^p_{\mathrm{mol}}(T)$ is the heat capacity. $K(T)$ can be evaluated from ref. [3] to be around 44 GPa, with $V_{\mathrm{mol}}(T) = 82.77\ \mathrm{cm}^3/\mathrm{mol}$ and phonon heat capacity values extracted from ref. [4], the γ_{macro} value asymptotically reaches $\sim$1 at higher temperature, which is overall consistent with the fitted γ_i since γ_{macro} is averaged over all phonon modes with different contributions, as well as the quasi-2D nature of $CrGeTe_3$ gives rise to more variations of phonon characters.

IV. Additional data and plots

Detailed field dependence. Fig.S1 shows the magnetic field dependence of the Raman signals from the 633nm measurements.

Detailed temperature dependence. Fig.S2 shows the raw data of the temperature dependence of the Raman signals from the 633nm and 532nm measurements. Here an alternative way to extract E_{avg} is used where we fit the whole spectrum with a single Gaussian peak, and its center value is taken as E_{avg} (Fig.S2 a,b). Equation (1) of the main text is used to fit the extracted E_{avg} as a function of temperature, where it gives $E(0)$=297.87 cm^{-1}, γ=2.07 for the 0$\sim$300K fit, and $E(0)$=297.88 cm^{-1}, γ=2.07 for the 65$\sim$300K fit (Fig.S2d).

Fig. S3 shows the frequency comb peak fits on the 633nm data set, where individial peaks are extracted using equal-width Voigt fits (Fig.S3a). The overall temperature dependence is similar to that of 532nm data set, and at least six peaks can be resolved in the fits below 70K, and four peaks can be resolved above (Fig.S3b). The temperature dependence fits for range [11, 300]K gives $E(0)$=298.26 cm^{-1}, γ=2.09, Γ_0 = 0.78 cm^{-1}, b = 2.12 cm^{-1}, and the [65, 300K]fit gives $E(0)$=298.20 cm^{-1}, γ=1.92, Γ_0 = 0.88 cm^{-1}, b =1.97 cm^{-1}.

Fig. S4 shows the frequency comb peak fits on the $CrSiTe_3$ A_g^5 phonons. The fitting on the highest energy mode gives Γ_0 = 0.47 cm^{-1}, b = 2.72 cm^{-1}. Notably, the FWHM of the highest mode is as low as 0.43cm^{-1}, close to the energy step size of 0.35cm^{-1}. This not only demonstrates the excellent energy resolution of the Raman setup but also indicates minimal decay associated with this phonon mode at low temperatures. The phonon softening arising from thermal expansion in $CrSiTe_3$ is difficult to estimate accurately due to the absence of systematic volumetric thermal expansion data. Nevertheless, the thermal expansion coefficient along the c-axis has been measured between 20 and 140 K [5], allowing for a reasonable extrapolation of α_c (Fig. S4b). In Fig. S4c, we compare the extrapolated c-axis expansion to the temperature dependence of the phonon energy using $E(0) = 518.44$ cm^{-1} and $\gamma = 0.17$, assuming a volumetric thermal expansion coefficient of $\beta = 3\alpha_c$. For reference, from previous measurements we can estimate the asymptotic value of the macroscopic Grüneisen parameter γ_{macro} in $CrSiTe_3$ to be approximately 1 [6, 7]. This discrepancy may arise from the fact that, in $CrXTe_3$ compounds, the c-axis contributes predominantly to volumetric expansion due to spin-lattice coupling-induced negative thermal expansion in the a and b directions. Moreover, the high energy of this phonon mode may result in a smaller contribution to thermal expansion overall.

References

[1] Tian, Y., Gray, M. J., Ji, H., Cava, R. J. & Burch, K. S. Magneto-elastic coupling in a potential ferromagnetic 2D atomic crystal. *2D Mater.* **3**, 025035 (2016).

[2] Carteaux, V., Brunet, D., Ouvrard, G. & André, G. Crystallographic, magnetic and electronic structures of a new layered ferromagnetic compound $Cr_2Ge_2Te_6$. *J. Phys. Condens. Matter* **7**, 69 (1995).

[3] Yu, Z., Xia, W., Xu, K., Xu, M., Wang, H. *et al.* Pressure-induced structural phase transition and a special resistivity plateau in $CrSiTe_3$. *J. Phys. Chem. C* **123**, 13885–13891 (2019).

[4] Lin, G. T., Zhuang, H. L., Luo, X., Liu, B. J., Chen, F. C. *et al.* Tricritical behavior of the two-dimensional intrinsically ferromagnetic semiconductor $CrSiTe_3$. *Phys. Rev. B* **95**, 245212 (2017).

[5] Ron, A., Zoghlin, E., Balents, L., Wilson, S. D. & Hsieh, D. Dimensional crossover in a layered ferromagnet detected by spin correlation driven distortions. *Nat. Commun.* **10**, 1654 (2019).

[6] Gohil, S., Halder, S., Iyer, K. K., Ghosh, S., Thamizhavel, A. *et al.* Magnetic order, field-induced melting, and role of spin-lattice coupling in two-dimensional van der Waals materials: A case study of $CrSiTe_3$. *Phys. Rev. B* **111**, L100407 (2025).

[7] Xu, K., Yu, Z., Xia, W., Xu, M., Mai, X. *et al.* Unique 2D–3D structure transformations in trichalcogenide $CrSiTe_3$ under high pressure. *J. Phys. Chem. C* **124**, 15600–15606 (2020).

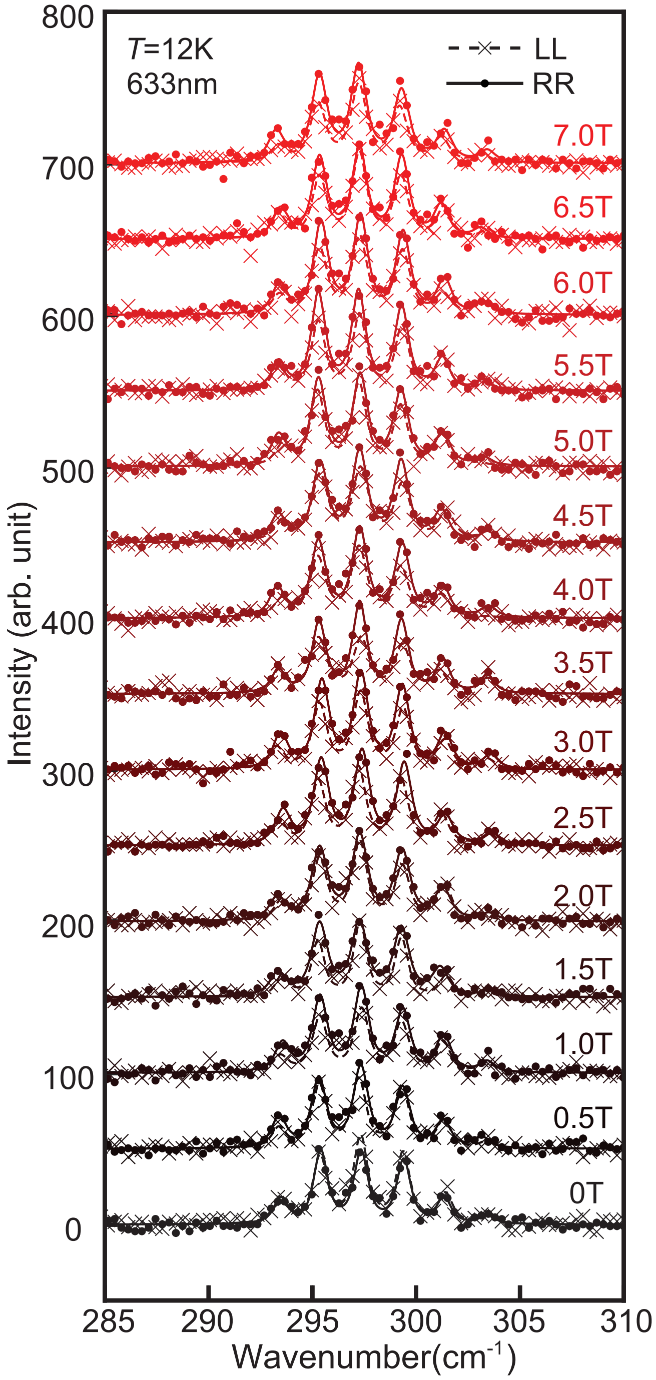

Figure S1: Detailed field dependence of the A_g^5 phonon mode. The dashed and solid lines represent voigt fits to the LL and RR polarizations, respectively.

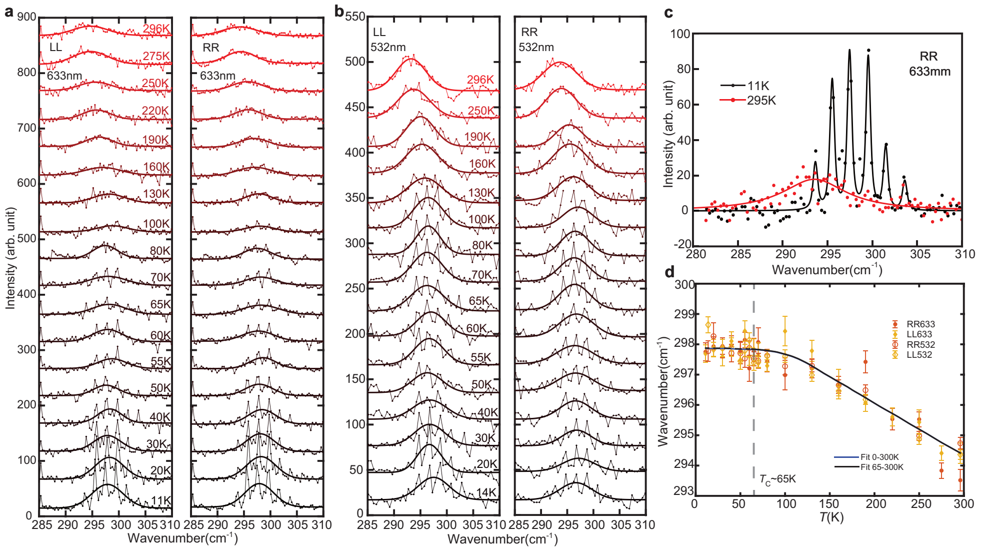

Figure S2: Detailed temperature dependence of the A_g^5 mode with one-peak Gaussian fits (solid lines) for (a) 633nm data and (b) 532nm data. The missing data points at 100K in (b) indicate spurious signals whose values are beyond the vertical scope of this plot. (c) A comparison of the A_g^5 mode between 11K and 295K. (d) Fitting of the temperature dependence using the single Gaussian peak fits from (a) and (b) using thermal expansion data. The blue solid line shows the fitting with the whole dataset, while the black line shows the fitting with only $T > 65$K data. Error bars in panel (d) represent one standard deviation.

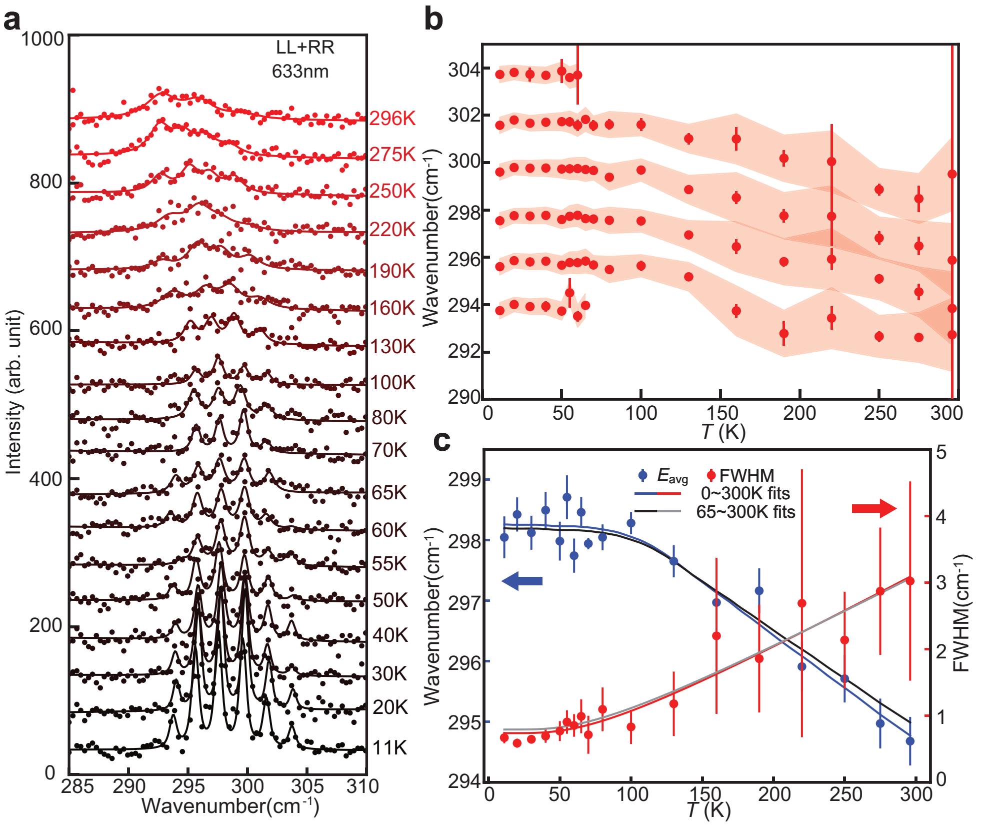

Figure S3: (a)Detailed temperature dependence of the A_g^5 mode from 633nm-laser experiment. Solid lines are Voigt fits. (b) Temperature dependence of the peak positions (red dots) and FWHMs (red shading) of the fitting result in (a). (c) Fitting of the temperature dependence of the energy "weighted average" using thermal expansion data and FWHMs using eqn.(1) in the main text, the blue and red solid lines show the fitting with the whole dataset, while the black and gray lines shows the fitting with only $T \geq 65$K data. Error bars in panels (b) and (c) represent one standard deviation.

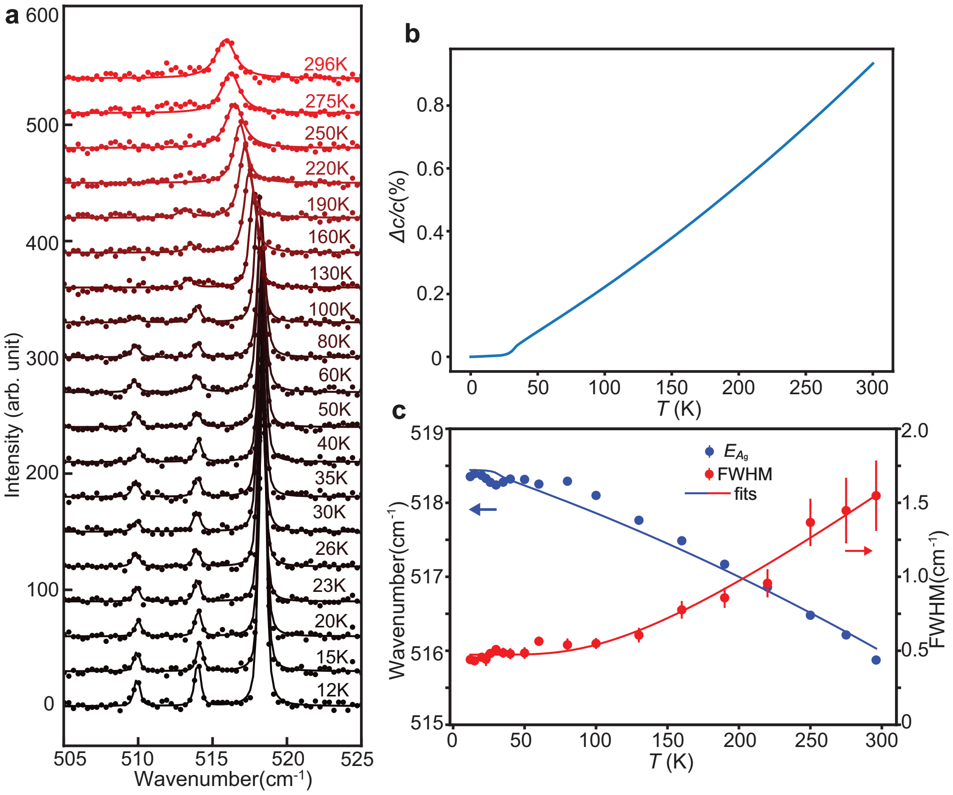

Figure S 4: (a) Fits on individual frequency comb peaks at different temperatures in $CrSiTe_3$. (b)Estimated thermal expansion of the c-axis based on Ref. [5] (adapted from Ron, A., Zoghlin, E., Balents, L. *et al.* Dimensional crossover in a layered ferromagnet detected by spin correlation driven distortions. *Nat Commun* **10**, 1654 (2019). https://doi.org/10.1038/s41467-019-09663-3). The 140–300 K range is obtained by linearly extrapolating the thermal expansion coefficient α_c. (c) Temperature dependence of the energy and FWHM of the highest peak. The red solid line is a fit according to eqn.(1) in the main text, while the blue solid line shows the simulated phonon softening due to c-axis thermal expansion. Error bars in panel (c) represent one standard deviation.
